# Perceived Parenting and Parent-Child Relational Qualities in Fathers and Mothers: Longitudinal Findings Based on Hong Kong Adolescents

**DOI:** 10.3390/ijerph17114083

**Published:** 2020-06-08

**Authors:** Daniel T. L. Shek, Diya Dou

**Affiliations:** Department of Applied Social Sciences, The Hong Kong Polytechnic University, Hong Kong, China; diya.dou@polyu.edu.hk

**Keywords:** parenting, parental control, parent-child relational quality, family environment, Chinese parents, adolescents

## Abstract

To understand how family environment and functioning change over time during adolescence, this study examined the developmental trajectories of perceived parent-child subsystem qualities indexed by parental control and parent-child relational qualities, and the related perceived differences between fathers and mothers. Longitudinal data were collected from 2023 students in 28 high schools in Hong Kong. Among the 28 schools, five schools were in Hong Kong Island, seven in Kowloon district, and 16 in New Territories. Students were invited to respond to measures of perceived parent-child subsystem qualities in six consecutive high school years from the 2009/10 academic year. Individual Growth Curve analyses and paired *t*-tests were used to explore the developmental trajectories of research variables and the differences between fathers and mothers. While parental behavioral control and psychological control generally declined throughout the high school years, parent-child relational quality showed a U-shaped trajectory. Parent gender significantly predicted the initial levels of all measures and changes in behavioral control and parent-child relational quality. Mothers showed higher levels of parental control and parent-child relational quality than did fathers at each time point. However, mothers showed a faster decrease in these measures than did fathers.

## 1. Introduction

Adolescence is a developmental period that many physical, psychological, emotional and social changes take place. In addition to challenges to physical and intellectual growth, searching for identity is an important task for adolescents [1]. Adolescents gradually become more independent, seek more autonomy, and establish their self-concepts and values, which lead to changes in family environment [2,3].

Entering adolescence brings challenges to both adolescents and their parents. Adolescents may consider their parents harsher and more controlling, while parents may see children rebellious and more irresponsible. Both parents and adolescents face the challenges of reorganizing responsibilities and establishing a more egalitarian family relationship [4]. These considerations suggest that the patterns and forms of parenting, as well as adolescents’ perceptions of parenting, would change over time when children become more independent [5]. In the process of relationship restructuring, an increase in conflicts and a decrease in closeness between parents and children might exist [2,6]. Unfortunately, there is still very limited longitudinal research exploring the development of parent-child subsystem qualities such as parental control and parent-child relationships over the whole course of adolescence [7]. Moreover, given that mothers and fathers may use different parenting strategies during adolescence, there is a need to further examine parental gender differences in parenting [4].

### 1.1. Development of Parent-Child Subsystem Qualities

Parent-child subsystem qualities play vital roles in adolescent development. In this study, we focused on parental control and parent-child relationship quality. Existing research has identified behavioral control and psychological control as two distinct dimensions of parental control [8,9]. Behavioral control pertains to parents’ use of rules and disciplines to directly regulate adolescent behavior, such as setting boundaries and monitoring adolescents’ activities [10]. It is generally considered a protective factor against adolescent problem behaviors [11]. Empirical studies have provided consistent findings that parental behavioral control declines over time during adolescence [12,13]. For example, in Chen, Liu and Li’s two-year study [12], 258 Chinese high school students perceived significantly lower levels of behavioral control when they grew older. Similar results were found in Keijsers and Poulin’s study [14], which revealed a decrease in perceived parental control in adolescents aged 14 to 19. Besides, the desire for increased autonomy leads to emotional detachment and separation from parents because children have less communication with parents or show less willingness to disclose themselves to parents [14,15]. Consequently, parents may encounter more difficulties in monitoring their children’s personal lives, and their knowledge about their children diminishes steadily [16,17]. When parents find behavioral control becomes less effective in regulating adolescents’ behaviors, they may reduce the use of this strategy [14].

Psychological control refers to parenting behaviors manipulating adolescents’ emotions, feelings, and thoughts using tactics such as love withdrawal, guilt induction, and shaming [9]. Existing research has revealed that psychological control hinders adolescent development and positively predicts both internalizing and externalizing problems, such as depression and delinquency [11,18,19,20]. Some researchers argued that adolescents may perceive psychological control to intensify in adolescence because a greater sense of self makes them more sensitive to parents’ behaviors that potentially violate their independence [18]. Parents may increasingly adjust psychological control when they perceive losing direct control of adolescent behavior [21]. However, empirical studies have shown a mixed picture [21,22]. Some empirical studies found a stable trend of psychological control from early to middle adolescence: Luyckx’s study [23] investigating Belgian students aged from 18 to 21 revealed that the level of psychological control was low and stable; Smetana and Daddis’ [20] two-year study examined 93 American students in early adolescence and found no clear relationship between adolescents’ age and the level of parental psychological control. However, Desjardins and Leadbeater’s study [24] based on Belgian 19–25-year-olds students revealed a decline in parental psychological control. Theoretically, as the use of psychological control strategy frustrates adolescents’ autonomy needs and intensifies parent-adolescent conflict, parents may reduce the use of this strategy to avoid potential conflicts and resentment from children. Hence, it can be hypothesized that parental psychological control decreases as adolescents move to adulthood [25].

Parent-child relational quality is another important factor influencing adolescent development. Although not inevitable, the relationship between parents and children often deteriorates when adolescents become independent [6]. This can be attributed to an increase in parent-child conflicts and a decrease in parental support when children move into adolescence [26]. To date, parent-child relationship has been regarded as showing a quadratic (U-shaped) pattern over the whole course of adolescence [5]. For example, the results of Whiteman et al.’s ten-year longitudinal study on family relationships [27] revealed that the developmental trajectory of parent-child intimacy showed U-shaped patterns over time. Similarly, Shanahan and colleagues’ study [6] described an increase in conflict frequency from early adolescence and then a decrease after middle adolescence. There is clear empirical evidence that the parent-child relational quality temporarily declines from early to middle adolescence [7,28,29]. When both parents and adolescents can appropriately define boundaries and responsibilities and settle issues of autonomy and independence, the relationship between parents and children can be gradually restored and move to an egalitarian pattern in late adolescence [7,14].

### 1.2. Parental Gender Differences

It is argued that fathers and mothers provide different socialization experiences for adolescents [30]. Compared to fathers, mothers tend to be more involved in parenting, more caring about children, and show higher levels of acceptance, warmth and support to children [31,32]. Mothers were also often found to be more responsive and sacrificial in parenting [33]. However, researchers found that mothers also present higher levels of parental control and rejection, set harsher discipline on children [34,35,36]. The high levels of responsiveness and control of mothers have a mixed impact on mother-child relationship. On the one hand, mothers were reported to have better communication and a more positive relationship with adolescents than were fathers [37]. On the other hand, mothers were also perceived by children to have more parent-child conflicts than were fathers [3].

In traditional Chinese society, fathers are often the harsher ones regulating children through strict disciplines, while mothers are more caring and kind. However, recent research on parenting in the Chinese context revealed that the roles of Chinese mothers and fathers have reversed, where mothers become the more controlling ones monitoring adolescents’ behaviors [36]. For example, Shek and colleagues’ cross-sectional survey conducted with adolescents in Hong Kong revealed that mothers in Hong Kong were perceived to be more demanding and harsher than were fathers, indicating that perceived maternal control was significantly higher than perceived paternal control [35,38]. However, more longitudinal data in this area are needed.

### 1.3. Research Gaps

The first research gap concerns a lack of longitudinal studies on the development of parenting and parent-child relational qualities during adolescence. Most available studies investigating parent-child relationships are based on cross-sectional data [7]. Given that cross-sectional research is problematic in informing cause-and-effect relationships, longitudinal research is needed to gain a deeper understanding of changes in parent-child subsystem qualities during adolescence.

The second research gap is that there is limited research on this topic covering the high school years (i.e., from early adolescence to late adolescence). Rogers et al. [21] argued that most available research has focused on early adolescence and very limited research has been conducted in late adolescence. Investigation of the development of parent-child relational qualities in both early adolescence and late adolescence is necessary for a comprehensive understanding of parenting in adolescent development [39].

The third research gap is that very few studies have included multiple measures of the parent-child subsystem quality. Particularly, although some longitudinal studies have examined parent support, conflicts [7], behavioral control [4], parents’ worry and over-control [40], longitudinal research on the development of psychological control remains limited [18]. In addition, adolescents’ satisfaction with parental control, which describes whether adolescents see the levels of parental control as reasonable, should be considered an indicator of parent-child relational quality [41]. However, this measure has been largely neglected in studies evaluating parent-child relational quality [42]. Hence, there is a need to use multiple measures in the assessment of parent-child relational quality.

The fourth research gap is that very few studies on the development of parenting and parent-child relational qualities have been conducted in different Chinese societies [33,43], except some longitudinal studies such as Shek’s work conducted in Hong Kong [36,41], and Liu’s work conducted in mainland China [44,45]. According to Bronfenbrenner’s ecological model [46], external influence and environment such as social policies and cultural values have a reciprocal relationship with children’s development. Parenting styles, patterns, and ideology can be vastly different across cultures, which significantly affect how parents practice their parenting behaviors, and eventually affect child developmental outcomes [47]. For example, Shek [36] argued that mothers’ role in daily caregiving in Chinese culture remained significant despite the increasing paternal involvement in Western societies. The parenting style of Asians may have a different outlook on the characteristics of authoritarian or controlling parenting, which suggests different cultural meanings. For example, although psychological control is believed to hinder adolescent development, some scholars argued that in Asian countries, the destructive consequences of parental psychological control may be mitigated by social cultures emphasizing family obligations and interdependence [48]. Interestingly, although “tiger parenting” has been used in the literature to portray Chinese parents who have extremely harsh discipline and emphasize academic achievement [49], a cross-cultural study revealed that Chinese adolescents consistently reported the lowest levels of parental control among thirteen cultural groups across the world [50]. Thus, more empirical studies conducted in the Chinese context are needed to enrich the indigenous scientific knowledge and our understanding of family functioning and parenting styles of Chinese parents. Using six waves of longitudinal data, this study examined the developmental trajectories of perceived parental control and parent-child relational quality during adolescence, and the differences between fathers and mothers in the related domains.

### 1.4. Research Questions in the Present Study

The first question is “what are the developmental trajectories of parent-child relational system qualities (behavioral control, psychological control, and parent-child relational quality) over the high school years?” Adolescents’ search for increase autonomy would lead to a drop in behavioral control from parents [13]. Different understandings of autonomy from parents and adolescents can promote conflicts and a decrease in parental support, which may distance adolescents from their parents and impair the parent-child relationship [12,51]. Despite the limited and inconclusive research findings on the development of psychological control during adolescence [21], a decrease of psychological control is observed in research focusing on the whole course of adolescence [24,25]. An explanation is that parents may gradually reduce the use of psychological control because the tactics such as love withdrawal and guilt induction would exert a negative influence on adolescent psychological development and even raise resentment from children [18]. In view of the gradual maturation of adolescents, we would expect a decrease in behavioral control (H1) and psychological control (H2) but a U-shaped developmental trajectory of parent-child relational quality (H3) from early to late adolescence.

The second question is “what are the differences between mothers and fathers in terms of parental behavioral control, psychological control and parent-child relational quality across the high school years?” As discussed earlier, previous studies generally demonstrated that mothers were more involved in parenting and tended to have stronger levels of control than did fathers during adolescence [4]. Compared to fathers, mothers were also found to be more supportive and manage the parent-child relationship better [7]. In line with the existing research findings [36], we hypothesized that mothers would be perceived as showing stronger behavioral control (H4), stronger psychological control (H5), and better parent-child relational quality (H6) than did fathers at each wave.

The present study also took student gender and initial age into account. Specifically, previous research has found that parents tended to exercise greater control over girls than boys or more likely to monitor daughters than sons, which may lead to different levels of parent-child subsystem qualities perceived by girls and boys [52]. As discussed earlier, previous studies revealed that different aspects of parental control declined from early to late adolescence whereas parent-child conflicts increased [7]. Thus, gender and initial age of the students were included in the present study as control variables.

## 2. Materials and Methods

### 2.1. Procedures and Participants

The present study was a part of a large-scale longitudinal study on the development of Chinese adolescents conducted in 28 high schools in Hong Kong. The first of the six-wave data collection took place in the 2009/10 academic year, with follow-ups in about a one-year interval. Among the 28 schools, five schools were located in Hong Kong Island, seven in Kowloon district, and 16 in New Territories. All grade 7 students from participating schools were invited to complete the same paper-and-pencil questionnaire for six consecutive years. The questionnaire aimed to evaluate students’ youth development, adjustment, and their parents’ parenting during the high school years. Trained research staff provided clear instructions to students and administrated the survey in classrooms during school hours. All participating students and their parents were informed of the aims of the study, the principles of voluntary participation, anonymity and data confidentiality. The project was evaluated and approved by the Human Subjects Ethics Sub-Committee (or its Delegate) at The Hong Kong Polytechnic University. Written consent forms were obtained from participating students, their parents and schools before the data collection.

In total, 3328 students completed the survey at Wave 1. As shown in Table 1, the number of participants at Wave 2 to Wave 6 indicates the number of students who have participated in the survey at current and all previous waves. For example, among 3328 students who completed the survey at Wave 1, 2905 students further participated in the survey at Wave 2 (attrition rate = 12.7%). Among these students, 2669 students joined the data collection at Wave 3 (attrition rate = 8.12% based on the sample size at Wave 2). The attrition rates ranged from 7.46% to 12.7% across the six waves. The matched sample (*N* = 2023) with participants who completed the survey at all of the six waves was used in the present study.

The sample inclusion criteria included student and parental consent and students’ full ability to understand written Chinese. Table 2 shows the sample characteristics at Wave 1.

In total, 2023 students (*M*_age_ at Wave 1 = 12.53 years, *SD*_age_ = 0.66) from 28 schools responded to the survey at all six waves, including 959 boys (47.4%), 1040 girls (51.4%) and 24 students (1.2%) who did not report their gender at Wave 1. Most of the students were from intact families (*N* = 1788, 88.4%). The 217 students (10.7%) reported that their parents were divorced, separated, or widowed at Wave 1.

### 2.2. Measures

The Parent-Child Subsystem Quality Scale (PCSQS) was used in the present study to evaluate the three parent-child subsystem qualities perceived by adolescents. This 17-item scale has shown to be a reliable and valid instrument possessing good psychometric properties [53,54]. The scale consists of three measures. The first is maternal/paternal behavioral control, including seven questions about students’ perceptions of their mother’s/father’s knowledge about the child, expectation, regulations, and monitoring. Some sample questions include “my father/mother asks me about what I did after school”, “my father/mother expects me to have good behavior in school”, and “my father/mother actively understands my afterschool activities”. The second measure is maternal/paternal psychological control, formed by four items tapping parental behaviors negatively affecting adolescents’ psychological world. Sample items include “my father/mother wants to change my thoughts”, “my father/mother thinks that his/her thoughts are more important than my thoughts”, and “my father/mother always wants to change me to fit his/her standards”. The third measure is mother-/father-child relational quality, indicated by the extent how the student is satisfied with parental control and active communication with the parent. This measure is formed by six items, such as “my father’s/mother’s discipline of me is reasonable”, “I am satisfied with the relationship between my father/mother and me”, and “I shared my feelings with my father/mother”. Students indicated the level of agreement to the statements on a 4-point Likert scale (1 = “strongly disagree,” 4 = “strongly agree”). In total, students were invited to respond to 17 items on paternal parenting and the same 17 items on maternal parenting. The average scores of each measure were calculated, respectively. Higher values of the average scores indicated stronger levels of parental control or better parent-child relational quality. Internal consistencies were high with Cronbach’s αs ranging between 0.87 to 0.89 for behavioral control, between 0.79 and 0.91 for psychological control and between 0.89 and 0.90 for parent-child relational quality across the six waves (see Table A1 in Appendix A). The mean inter-item correlations were above 0.49 for behavioral control, above 0.48 for psychological control and above 0.58 for parent-child relational quality.

### 2.3. Analysis Plan

To examine the developmental trajectories of parental control and parent-child relational quality, we used individual growth curve (IGC) analyses to explore the interaction effect of time (Wave 1–6) and perceived parental behavior (fathers versus mothers). Before conducting IGC analyses, we adjusted the original format of our data for a repeated-measures design. The original data were in a format that each row represented a student, and each repeated-measures variable of each parent (e.g., maternal behavioral control) collected at six time points was represented by six different columns. For a mixed model, we need the variable time to be represented by a single column. In addition, as the data of mothers and fathers were based on adolescent reports and thus inherently non-independent, parent gender was treated as a within-subject predictor. Thus, we further transposed the data and used three columns to represent the three parent-child subsystem qualities. In the final data set, each student is represented by 12 rows (each for the perception of one parent at one time point). Invariant variables, including child gender and initial age, have the same values within each student.

A series of IGC models were established for the three parent-child subsystem qualities, respectively. For each parent-child subsystem quality measure, we first set up an unconditional mean model serving as a baseline model to estimate individual variation in each research variable without taking time into account. To evaluate the extent to which between-individual differences contributed to the amount of total variation in the research variables, we calculated the intraclass correlation coefficient (ICC). The six waves time points were coded accordingly (i.e., Wave 1 = 0, Wave 2 = 1, Wave 3 = 2, Wave 4 = 3, Wave 5 = 4, Wave 6 = 4.83). Then we gradually added linear (i.e., time) and quadratic (i.e., time^2^) growth parameters into each model to explore non-linear development of the parent-child subsystem quality variables. Models were selected by evaluating whether a linear or quadratic change would better capture the developmental trajectories. Next, we added parent gender into the model to test whether it would predict the growth parameters, including initial status, linear and quadratic changes, in the three parent-child subsystem qualities across time. The effects of student gender and initial age were controlled when examining the relationships among research variables. We allowed both the intercept and linear slope to vary across individuals. Parent gender and student gender were dummy coded (“father” = “−1”, “mother” = “1”; “boy” = “−1”, “girl” = “1”). The equation defining the 2-level model is displayed in Appendix B. The indices we used to index model fit included “−2log likelihood,” “Akaike Information Criterion” (AIC), and “Bayesian Information Criterion” (BIC). A better model fit is achieved when the values of these indices decrease.

To examine parental differences in parent-child subsystem qualities, we looked at the interaction between parents (fathers versus mothers) and time (Wave 1 to Wave 6). In addition, we compared parent-child subsystem qualities between mothers and fathers at each wave separately through paired *t*-tests as the data of both parents relied on adolescents’ reports. All data analyses were conducted using SPSS (version 25.0, IBM Corp, Armonk, NY, USA).

## 3. Results

### 3.1. Descriptions and Correlations among Variables

The descriptive statistics and the results of correlation analysis for the related measures are shown in Table A1 and Table A2 in Appendix A, respectively. The results indicated that the three paternal variables were all significantly correlated with each other at all waves. Similar correlations were also found between maternal variables except for Wave 6 when maternal behavioral control was uncorrelated with maternal psychological control. In addition, the parent-child relational quality of a parent was positively correlated with the behavioral control of that parent and was negatively correlated with the psychological control of that parent. Besides, significant correlations were found in each pair of maternal and paternal variables.

### 3.2. Perception of the Parent-Child Subsystem Qualities over Time

Three unconditional models, Model 1a, Model 1b and Model 1c, were established for the three parent-child subsystem qualities, respectively. The values of ICC of behavioral control, psychological control and parent-child relational quality were 0.355, 0.356, and 0.396, indicating that 35.5%, 35.6%, and 39.6% of the variance in the three variables were attributable to individual differences. Thus, both Level-1 and Level-2 parameters were included in IGC models for the three parent-child subsystem qualities.

As shown in Table 3, Table 4 and Table 5, when time was added, Model 2a, Model 2b and Model 2c achieved better model performance compared to Model 1a (∆χ^2^ (3) = 380.020, *p* < 0.01, ∆AIC = 374.020, ∆BIC = 349.788), Model 1b (∆χ^2^ (3) = 535.369, *p* < 0.01, ∆AIC = 529.369, ∆BIC = 505.140) and Model 1c (∆χ^2^ (3) = 513.300, *p* < 0.01, ∆AIC = 507.300, ∆BIC = 483.070), respectively, suggesting that time significantly explained the variances in all the three research variables. When a quadratic time slope was further included, the model fit of Model 3c was significantly improved than that of Model 2c (∆χ^2^ (1) = 19.804, *p* < 0.01, ∆AIC = 17.804, ∆BIC = 9.727), while Model 3a and Model 3b failed to fit the data better than did Model 2a (∆χ^2^ (1) = 5.357, *p* < 0.05, ∆AIC = 3.353, ∆BIC = −4.725) and Model 2b (∆χ^2^ (1) = 1.484, *p* > 0.05, ∆AIC = −0.156, ∆BIC = −8.592), respectively. Thus, the quadratic slope was only included in the final model of parent-child relational quality but not in the final models of parental behavioral and psychological control. The results indicated that both behavioral and psychological control showed a linear decline over time (*β* = −0.029, *p* < 0.001; *β* = −0.018, *p* < 0.001, respectively). As specified in Table 5, parent-child relational quality decreased over the high school years (*β* = −0.050, *p* < 0.001) and then slightly increased (*β* = 0.006, *p* < 0.001) over time, presenting a U-shaped trajectory. In short, Hypotheses 1 to 3 were supported.

### 3.3. Parent Gender on Initial Levels of Parent-Child Subsystem Qualities

As shown in Table 6, results demonstrated that parent gender yielded significant predictive effect on the initial levels of behavioral control (*β* = 0.229, *p* < 0.001), psychological control (*β* = 0.022, *p* < 0.001), and parent-child relational quality (*β* = 0.125, *p* < 0.001). The results of IGC showed that mothers were perceived to have stronger behavioral control, stronger psychological control and better parent-child relational quality than were fathers at Wave 1. Student gender significantly accounted for the variability in the intercept of psychological control (*β* = −0.075, *p* < 0.001), indicating that girls reported a lower level of psychological control than did boys at Wave 1.

### 3.4. Parent Gender on the Change Rate of Parent-Child Subsystem Qualities over Time

**Figure 1:**
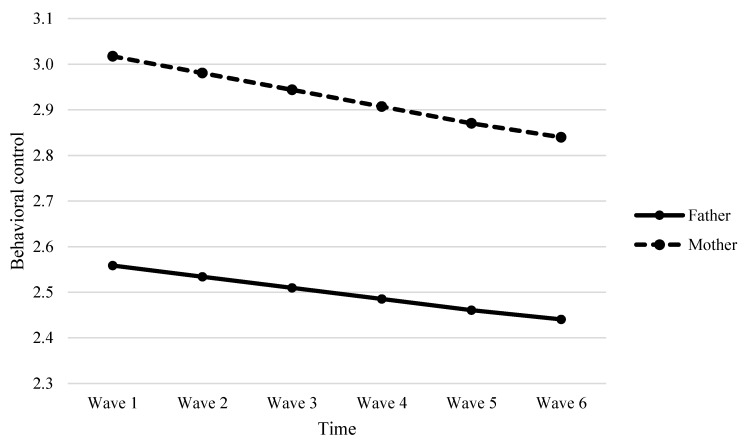
Fitted trajectories of behavioral control of mothers and fathers. The figures were plotted based on Model 4a shown in Table 6.

**Figure 2:**
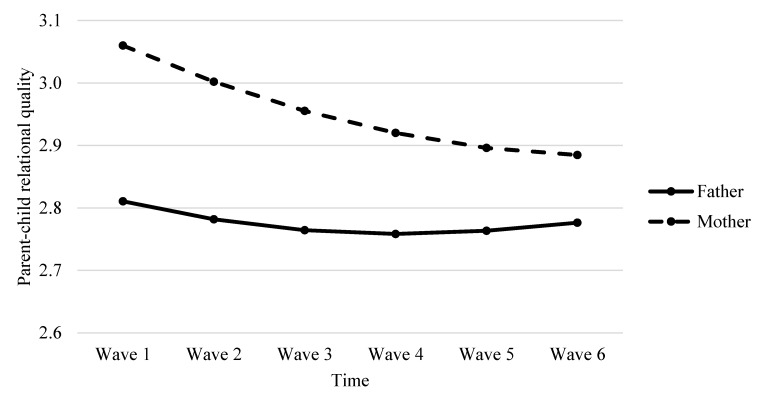
Fitted trajectories of parent-child relational quality of mothers and fathers. The figures were plotted based on Model 4c shown in Table 6.

**Figure 3:**
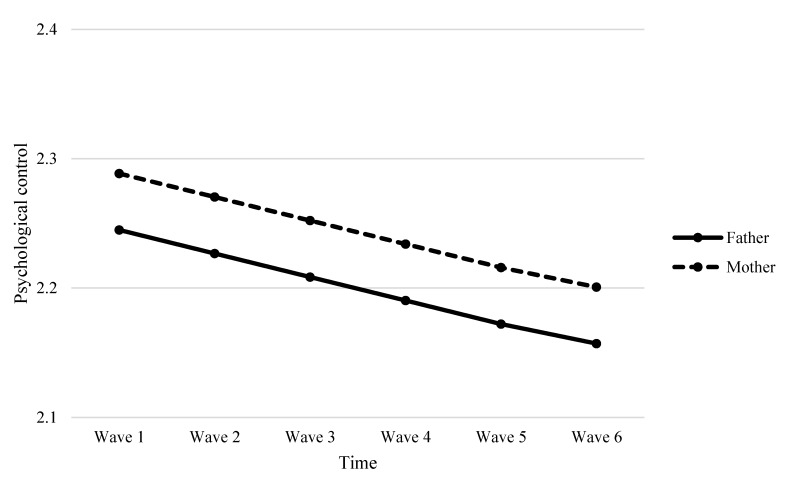
Fitted trajectories of psychological control of mothers and fathers. The figures were plotted based on Model 4b shown in Table 6.

As indicated in Table 6, results showed that parent gender was a significant predictor of variability in the linear change in behavioral control (*β* = −0.006, *p* < 0.001, see Model 4a) and parent-child relational quality (*β* = −0.015, *p* < 0.05, see Model 4c). The negative direction of predictive effects indicated that mothers demonstrated a faster decrease in behavioral control and parent-child relational quality than did fathers over time (see Figure 1 and Figure 2). Interestingly, we observed a marginally significant parent gender effect on the quadratic slope for parent-child relational quality (*β* = 0.003, *p* = 0.054, see Model 4c in Table 6). As evident in Figure 2, mother-child relational quality decreased faster than father-child relational quality initially, but this decreasing trend for mothers was gradually slower compared to that for fathers over time. However, parent gender did not significantly predict the change rate of psychological control (*β* = −0.002, *p* > 0.05, see Model 4b in Table 6), meaning that the development of psychological control over time did not significantly differ between mothers and fathers. The developmental trajectories of maternal and paternal psychological control were depicted in Figure 3. The results also revealed student gender as a significant predictor of the linear slope of behavioral control (*β* = 0.005, *p* < 0.05), meaning that girls tended to perceive a slower decrease in behavioral control than boys.

### 3.5. Mean Comparisons between Maternal and Paternal Parenting at Each Time Point

As displayed in Table 7, the results of the paired *t*-tests showed that mothers were perceived to have significantly stronger behavioral control than fathers at all six waves (Cohen’s *d*s > 0.704). A similar pattern was observed in parent-child relational quality, although the effect sizes were relatively small (Cohen’s *d*s ranging between 0.299 to 0.368). As to psychological control, significant differences were observed at Wave 1, Wave 3, and Wave 5 with very small effect sizes (Cohen’s *d*s < 0.071). For the comparisons at Wave 2, Wave 4, and Wave 6, the means of psychological control of mothers were consistently higher than that of fathers (with no significant difference though), which to some degree provided support to Hypothesis 5. In short, Hypotheses 4 to 6 were generally supported.

## 4. Discussion

Using six waves of survey data collected from high school students in Hong Kong, this study set out to investigate the developmental trajectories and parent gender differences in parent-child subsystem qualities indexed by parental control and parent-child relational quality. This study has several unique features. First, we collected six waves of longitudinal data using a relatively large sample of adolescents (*N* = 2023) to better explain the development of parent-child relations and ensure the statistical power of analyses. Second, the focus of this study covered early adolescence to late adolescence across the high school years. Third, multiple indicators of parent-child subsystem qualities were used. In particular, we examined psychological control and included children’s satisfaction with parental control as an indicator of parent-child relational quality. Fourth, the study was conducted with adolescents in a Chinese context [55].

For the first research question, the results showed that parent-child subsystem qualities changed during adolescence for both adolescent boys and girls. The findings suggest that the family processes for adolescent boys and girls are fairly similar. The results indicated a linear decrease in parental behavioral control from early to late adolescence, which was in agreement with previous studies [12,36]. The literature on youth development has suggested that young people usually increase their need for autonomy striving to build social relationships and become independent young adults during adolescence [56]. Thus, the reduction of parental behavioral control will create more space where children can make their own decisions and develop self-concepts.

As to the development of psychological control, we observed a linear decline of parental psychological control during adolescence. In fact, among research exploring the changes in parental psychological control, different patterns were observed, including a stable [22,57], an increasing [58], a decreasing [20,24] and a potentially down U-shaped trajectory [21]. One possible explanation to our finding is that contemporary Chinese parents may gradually reduce the use of psychological control as they are increasingly aware of the negative influence of psychological control on children. Likewise, in Qin and colleagues’ study on parenting in Chinese American families [59], it was argued that although these parents wanted to exert high levels of control over children, they felt guilty and tried to help children to become more independent. This can be the case in Hong Kong, where some Western values, such as autonomy and independence, are highly respected. Another possible explanation is that the perceived levels of psychological control are subject to adolescents’ views on how their parents respond to independence-related developmental tasks [9]. According to Smetana and Daddis [20], adolescents may perceive the psychological control behaviors that did intrude their important self-identities more unwanted than those behaviors that did not. Considering that social norms may also shape adolescents’ perception [48], Chinese adolescents may be less sensitive to the negative impact of parental psychological control and thus report a decrease longitudinally as they become more independent [60].

Regarding the development of parent-child relational quality, we observed a U-shaped trajectory, suggesting a decline from early to middle adolescence and later an increase after middle adolescence. Our findings are consistent with previous research [7,28,29]. In particular, the parent-child relational quality was operationalized by active communication and adolescents’ satisfaction with parental control. As suggested by Qin and colleagues [59], some adolescents see parental control as an expression of responsibility and care. Therefore, they may feel emotionally neglected when parental control is absent. Bearing the social and contextual considerations in mind, our findings suggest that the temporary decrease in parent-child relational quality may be universal.

For the second research question, we found that mothers were generally perceived to have stronger behavioral and psychological control and better parent-child relational quality than were fathers. These findings echo previous work conducted with adolescents in Hong Kong [19,36,61]. This study also reinforces the conjecture that the roles of mothers and fathers in Chinese families substantially changed. The traditional family structure in Chinese families has set clear roles and responsibilities for men and women. In particular, men are expected to provide resources for the family and have all the power in leading the family forward. In contrast, women often play subordinate roles, taking care of children and the elderly at home. The pattern of traditional Chinese parenting is also reflected in a Chinese saying of “yan fu ci mu”, which means “strict father, kind mother”. Our results indicated that this pattern has changed to “strict mother, kind father”, which was in line with recent research on contemporary Chinese families [58]. In addition, we found that mothers demonstrated a faster decrease in behavioral control than fathers. It is possible that mothers adapt their parenting behaviors better during adolescence as they are actively involved and more sensitive to children’s needs and development [62]. Moreover, mother-child relational quality firstly presented a faster decrease and then a slower decrease than father-child relational quality over time. A possible explanation is that mothers’ involvement may be perceived as over-controlling in early adolescence [62]. For example, a study showed that mothers’ worry, but not fathers’ worry, tended to be perceived as overly controlled by adolescents [40]. Our results suggested that Chinese mothers seemed to be actively involved in parenting and meanwhile actively adapted their parenting behaviors to adolescent development in comparison with Chinese fathers. This finding supported the idea that mothers may be more authoritative than fathers in Chinese societies [36]. According to Shek [58], the relatively lower levels of fathers’ involvement can be attributed to economic and social factors such as heavy economic pressure in contemporary Hong Kong and the changing roles of fathers and mothers in parenting in the Chinese culture. In short, our results are consistent with earlier studies revealing differences in parent-child subsystem qualities between Chinese mothers and fathers. Both cross-sectional studies [63] and longitudinal studies [36,58] have underscored the central role of mothers in the parenting process in Chinese families.

As to child gender difference, the study revealed that girls reported a lower initial level of perceived psychological control than did boys, which was in line with previous research [36]. In addition, girls tended to perceive a slower decrease in behavioral control than did boys. As it is argued that Chinese girls tend to report a positive relationship with parents than do boys [36], girls may have a relatively positive view on behavioral control, which might slow the changing process of parental behavioral control. Moreover, as social norms in Chinese societies require females to be obedient, understanding and considerate, Chinese parents might also set more regulations and expect greater conformity and obedience for girls than for boys [64,65].

Despite the pioneering nature of the study, the present study has several limitations. The first limitation concerns the single-informant design. Although we believe that adolescents’ own perceptions can more accurately represent their experience [52], it would be informative to include parents’ perceptions of parent-child subsystem qualities [4,42]. The use of multi-informant data can minimize the drawbacks associated with a single informant [52]. Second, as maternal and paternal factors may reciprocally influence each other over time, longitudinal studies investigating the interaction between fathers’ and mothers’ parenting can enrich our understanding of the changing dynamic of family relations and the potential influence on adolescent development [33]. Third, detailed analyses concerning family intactness can be included in future studies as families with different structures may vary in family functioning and parenting [66]. Finally, it would be illuminating if other aspects of the parent-child relational quality, such as autonomy support, can be examined.

## 5. Conclusions

This study adds value to the existing literature by depicting the developmental trajectories of different aspects of parent-child subsystem qualities. Both behavioral control and psychosocial control of parents were perceived to decline across the adolescent years, while the parent-child relational quality showed a U-shaped developmental trajectory over time. This study offers insight into the changing dynamics of family environment and functioning across adolescence. As our findings suggested that fathers seemed to make adjustments in their parenting less actively than mothers, fathers may need to actively reorganize parenting responsibilities and promote establishing a more egalitarian family relationship. Besides, in this process of relationship restructuring, our data support the recommendation that parents should also pay attention to the enhancement of mutual trust and relationship between parents and adolescents.

## Figures and Tables

**Table 1:** The sample characteristics and sample size at each wave.

	Wave 1	Wave 2	Wave 3	Wave 4	Wave 5	Wave 6
*N* (Participants)	3328	2905	2669	2429	2186	2023
*M*_age_	12.59	13.59	14.54	15.49	16.37	17.20
*SD*_age_	0.74	0.70	0.68	0.66	0.63	0.58
Gender						
Male *N*	1719	1445	1318	1186	1049	974
Male %	51.7%	49.7%	49.4%	48.8%	48.0%	48.1%
Female *N*	1572	1419	1333	1234	1129	1044
Female %	47.2%	48.8%	49.9%	50.8%	51.6%	51.6%
Attrition rate (%)		12.7%	8.12%	8.99%	10.00%	7.46%

**Table 2:** The sample characteristics at Wave 1 (*N* = 2023).

Characteristic	*M*	*SD*	Frequency	Percentage
Student’s initial age	12.53	0.66		
Student gender				
Male			959	47.4%
Female			1040	51.4%
Family intactness				
Intact			1788	88.4%
Non-intact			217	10.7%

**Table 3:** Results of IGC models for parental behavioral control (Wave 1–6).

		Model 1a (Unconditional)		Model 2a (Linear)		Model 3a (Quadratic)	
		Estimate	SE	Estimate	SE	Estimate	SE
Fixed effects							
Intercept	β_0*j*_						
Intercept	γ_00_	2.718 ***	0.009	2.790 ***	0.011	2.800 ***	0.012
Linear Slope	β_1*j*_						
Time	γ_10_			−0.029 ***	0.002	−0.044 ***	0.007
Quadratic Slope	β_2*j*_						
Time^2^	γ_20_					0.003 *	0.001
Random effects	-						
Level 1 (within)	-						
Residual	r*_ij_*	0.253 ***	0.002	0.240 ***	0.002	0.240 ***	0.002
Level 2 (between)							
Intercept	u_0*j*_	0.139 ***	0.005	0.173 ***	0.008	0.173 ***	0.008
Time	u_1*j*_			0.003 ***	0.000	0.003 ***	0.000
Fit statistics							
Deviance		38,934.236		38,554.216		38,548.864	
Difference in deviance				Δ380.020 ***		Δ5.353 *	
AIC		38,940.236		38,566.216		38,562.864	
BIC		38,964.468		38,614.680		38,619.405	
Intra-class correlation		0.355					
df		3		6		7	

Note. AIC = Akaike Information Criterion; BIC = Bayesian Information Criterion. * *p* < 0.05. *** *p* < 0.001.

**Table 4:** Results of IGC models for parental psychological control (Wave 1–6).

		Model 1b (Unconditional)		Model 2b (Linear)		Model 3b (Quadratic)	
		Estimate	SE	Estimate	SE	Estimate	SE
Fixed effects							
Intercept	β_0*j*_						
Intercept	γ_00_	2.217 ***	0.010	2.261 ***	0.012	2.266 ***	0.013
Linear Slope	β_1*j*_						
Time	γ_20_			−0.018 ***	0.003	−0.027 ***	0.008
Random effects							
Level 1 (within)							
Residual	r*_ij_*	0.334 ***	0.003	0.307 ***	0.003	0.307 ***	0.003
Level 2 (between)							
Intercept	u_0*j*_	0.185 ***	0.007	0.230 ***	0.010	0.230 ***	0.010
Time	u_1*j*_			0.009 ***	0.001	0.009 ***	0.001
Fit statistics							
Deviance		45,481.027		44,945.658		44,944.174	
Difference in deviance				Δ535.369 ***		Δ1.484	
AIC		45,487.027		44,957.658		44,958.174	
BIC		45,511.256		45,006.115		45,014.707	
Intra-class correlation		0.356					
df		3		6		7	

Note. AIC = Akaike Information Criterion; BIC = Bayesian Information Criterion. *** *p* < 0.001.

**Table 5:** Results of IGC models for parent-child relational quality (Wave 1–6).

		Model 1c (Unconditional)		Model 2c (Linear)		Model 3c (Quadratic)	
		Estimate	SE	Estimate	SE	Estimate	SE
Fixed effects							
Intercept	β_0*j*_						
Intercept	γ_00_	2.869 ***	0.009	2.920 ***	0.012	2.939 ***	0.012
Linear Slope	β_1*j*_						
Time	γ_10_			−0.021 ***	0.002	−0.050 ***	0.007
Quadratic Slope	β_2*j*_						
Time^2^	γ_20_					0.006 ***	0.001
Random effects							
Level 1 (within)							
Residual	r*_ij_*	0.243 ***	0.002	0.225 ***	0.002	0.225 ***	0.002
Level 2 (between)							
Intercept	u_0*j*_	0.160 ***	0.006	0.220 ***	0.009	0.220 ***	0.009
Time	u_1*j*_			0.006 ***	0.000	0.006 ***	0.000
Fit statistics							
Deviance		38,227.216		37,713.916		37,694.112	
Difference in deviance				Δ513.300 ***		Δ19.804 ***	
AIC		38,233.216		37,725.916		37,708.112	
BIC		38,257.447		37,774.377		37,764.649	
Intra-class correlation		0.396					
df		3		6		7	

Note. AIC = Akaike Information Criterion; BIC = Bayesian Information Criterion. *** *p* < 0.001.

**Table 6:** Results of IGC models with level-2 predictors for parental behavioral control (Model 4a), psychological control (Model 4b) and parent-child relational quality (Model 4c).

		Model 4a (Behavioral Control)			Model 4b (Psychological Control)			Model 4c (Parent-Child Relational Quality)		
		Estimate	SE	*p*	Estimate	SE	*p*	Estimate	SE	*p*
Fixed effects										
Intercept	β_0*j*_									
Intercept	γ_00_	2.788 ***	0.011	0.000	2.267 ***	0.012	0.000	2.935 ***	0.013	0.000
Parent gender ^a^	γ_01_	0.229 ***	0.005	0.000	0.022 ***	0.007	0.001	0.125 ***	0.007	0.000
Student age	γ_02_	–0.023 *	0.011	0.031	0.007	0.012	0.563	–0.036 **	0.013	0.005
Student gender ^b^	γ_03_	–0.007	0.011	0.498	–0.075 ***	0.012	0.000	0.002	0.013	0.852
Linear Slope	β_1*j*_									
Time	γ_10_	–0.031 ***	0.002	0.000	–0.018 ***	0.003	0.000	–0.049 ***	0.007	0.000
Parent gender ^a^	γ_11_	–0.006 ***	0.002	0.000	–0.002	0.002	0.481	–0.015 *	0.007	0.026
Student age	γ_12_	0.001	0.002	0.804	–0.002	0.003	0.421	0.008	0.007	0.217
Student gender ^b^	γ_13_	0.005 *	0.002	0.023	0.003	0.003	0.311	0.006	0.007	0.405
Quadratic Slope	β_2*j*_									
Time^2^	γ_20_							0.006 ***	0.001	0.000
Parent gender ^a^	γ_21_							0.003	0.001	0.054
Student age	γ_22_							–0.001	0.001	0.601
Student gender ^b^	γ_23_							0.001	0.001	0.452
Random effects										
Level 1 (within)										
Residual	r*_ij_*	0.186 ***	0.002	0.000	0.308 ***	0.003	0.000	0.211 ***	0.002	0.000
Level 2 (between)										
Intercept	u_0*j*_	0.187 ***	0.008	0.000	0.224 ***	0.010	0.000	0.224 ***	0.009	0.000
Time	u_1*j*_	0.005 ***	0.000	0.000	0.009 ***	0.001	0.000	0.006 ***	0.000	0.000
Fit statistics										
Deviance		32,746.837			43,973.882			35,623.834		
AIC		32,770.837			43,997.882			35,655.834		
BIC		32,867.500			44,094.532			35,784.712		
df		12			12			16		

Note. ^a^ Father = –1, Mother = 1; ^b^ Boy = –1, Girl = 1; AIC = Akaike Information Criterion; BIC = Bayesian Information Criterion. * *p* < 0.05. ** *p* < 0.01. *** *p* < 0.001.

**Table 7:** Descriptive statistics and paired t-test results for parent-child subsystem qualities between fathers and mothers.

		Father		Mother		95% CI		*t*	*df*	*p*	Cohen’s *d*
		Mean	SD	Mean	SD	Lower	Upper				
BC	Wave 1	2.566	0.659	3.041	0.605	−0.505	−0.445	−31.156	1991	0.000	0.698
	Wave 2	2.534	0.631	2.980	0.583	−0.475	−0.417	−30.146	1952	0.000	0.682
	Wave 3	2.505	0.615	2.919	0.571	−0.442	−0.386	−29.114	1951	0.000	0.659
	Wave 4	2.501	0.594	2.920	0.548	−0.446	−0.392	−30.445	1927	0.000	0.693
	Wave 5	2.470	0.580	2.884	0.543	−0.440	−0.387	−30.478	1919	0.000	0.696
	Wave 6	2.453	0.589	2.860	0.523	−0.433	−0.382	−30.788	1913	0.000	0.704
PC	Wave 1	2.227	0.703	2.279	0.756	−0.084	−0.020	−3.151	1970	0.002	0.071
	Wave 2	2.249	0.708	2.273	0.745	−0.058	0.009	−1.419	1950	0.156	0.032
	Wave 3	2.186	0.725	2.229	0.742	−0.076	−0.009	−2.485	1948	0.013	0.056
	Wave 4	2.186	0.712	2.212	0.730	−0.059	0.007	−1.537	1926	0.124	0.035
	Wave 5	2.160	0.674	2.193	0.704	−0.065	0.000	−1.980	1918	0.048	0.045
	Wave 6	2.172	0.710	2.199	0.720	−0.059	0.006	−1.579	1914	0.114	0.036
RQ	Wave 1	2.815	0.690	3.072	0.655	−0.287	0.226	−16.367	1981	0.000	0.368
	Wave 2	2.780	0.676	2.986	0.642	−0.236	−0.175	−13.230	1952	0.000	0.299
	Wave 3	2.758	0.653	2.968	0.601	−0.239	−0.180	−13.828	1950	0.000	0.313
	Wave 4	2.748	0.633	2.960	0.570	−0.241	−0.183	−14.370	1926	0.000	0.327
	Wave 5	2.730	0.628	2.947	0.566	−0.246	−0.188	14.622	1918	0.000	0.334
	Wave 6	2.726	0.621	2.946	0.542	0.249	−0.191	−14.849	1914	0.000	0.339

Note: BC = Behavioral control; PC = Psychological control; RQ = Parent-child relational quality.

## Appendix A

**Table A1:** Reliability of scales and descriptive results of variables across the six waves.

Scale	Items	Wave	Cronbach’s α	Mean Inter-Item Correlation	Mean	SD
Father-child subsystem quality	17					
Paternal behavioral control	7	1	0.89	0.53	2.57	0.66
		2	0.88	0.52	2.53	0.63
		3	0.88	0.51	2.51	0.61
		4	0.88	0.51	2.50	0.59
		5	0.88	0.51	2.47	0.58
		6	0.89	0.53	2.45	0.59
Paternal psychological control	4	1	0.79	0.48	2.23	0.70
		2	0.83	0.54	2.25	0.71
		3	0.85	0.59	2.19	0.72
		4	0.86	0.61	2.19	0.71
		5	0.85	0.60	2.16	0.67
		6	0.88	0.65	2.17	0.71
Father-child relational quality	6	1	0.89	0.58	2.82	0.69
		2	0.90	0.61	2.78	0.68
		3	0.90	0.60	2.76	0.65
		4	0.90	0.61	2.75	0.63
		5	0.90	0.61	2.73	0.63
		6	0.90	0.62	2.73	0.62
Mother-child subsystem quality	17					
Maternal behavior control	7	1	0.89	0.53	3.04	0.61
		2	0.88	0.52	2.97	0.58
		3	0.88	0.52	2.92	0.57
		4	0.87	0.50	2.91	0.55
		5	0.88	0.51	2.87	0.55
		6	0.87	0.49	2.85	0.53
Maternal psychological control	4	1	0.85	0.58	2.28	0.76
		2	0.87	0.63	2.28	0.75
		3	0.88	0.66	2.23	0.74
		4	0.89	0.68	2.22	0.73
		5	0.90	0.68	2.20	0.70
		6	0.91	0.71	2.20	0.72
Mother-child relational quality	6	1	0.90	0.61	3.07	0.65
		2	0.90	0.61	2.99	0.64
		3	0.90	0.60	2.97	0.60
		4	0.89	0.59	2.96	0.57
		5	0.90	0.60	2.94	0.57
		6	0.89	0.59	2.94	0.55

**Table A2:** Correlations among research variables.

	Wave 1	Mean	SD	1	2	3	4	5	6
1	Paternal behavioral control	2.57	0.66	1.00					
2	Paternal psychological control	2.23	0.70	0.141 **	1.00				
3	Father-child relational quality	2.82	0.69	0.690 **	−0.107 **	1.00			
4	Maternal behavioral control	3.04	0.61	0.424 **	0.053 *	0.353 **	1.00		
5	Maternal psychological control	2.28	0.76	0.000	0.505 **	−0.094 **	0.084 **	1.00	
6	Mother-child relational quality	3.07	0.65	0.368 **	−0.030	0.463 **	0.671 **	−0.161 **	1.00
	**Wave 2**	**Mean**	**SD**	**1**	**2**	**3**	**4**	**5**	**6**
1	Paternal behavioral control	2.53	0.63	1.00					
2	Paternal psychological control	2.25	0.71	0.088 **	1.00				
3	Father-child relational quality	2.78	0.68	0.649 **	−0.188 **	1.00			
4	Maternal behavioral control	2.97	0.58	0.422 **	0.010	0.352 **	1.00		
5	Maternal psychological control	2.28	0.75	−0.010	0.465 **	−0.120 **	0.053 *	1.00	
6	Mother-child relational quality	2.99	0.64	0.335 **	−0.075 **	0.459 **	0.609 **	−0.276 **	1.00
	**Wave 3**	**Mean**	**SD**	**1**	**2**	**3**	**4**	**5**	**6**
1	Paternal behavioral control	2.51	0.61	1.00					
2	Paternal psychological control	2.19	0.72	0.111 **	1.00				
3	Father-child relational quality	2.76	0.65	0.670 **	−0.176 **	1.00			
4	Maternal behavioral control	2.92	0.57	0.440 **	0.030	0.337 **	1.00		
5	Maternal psychological control	2.23	0.74	−0.030	0.466 **	−0.145 **	0.054 *	1.00	
6	Mother-child relational quality	2.97	0.60	0.333 **	−0.070 **	0.432 **	0.608 **	−0.305 **	1.00
	**Wave 4**	**Mean**	**SD**	**1**	**2**	**3**	**4**	**5**	**6**
1	Paternal behavioral control	2.50	0.59	1.00					
2	Paternal psychological control	2.19	0.71	0.069 **	1.00				
3	Father-child relational quality	2.75	0.63	0.657 **	−0.237 **	1.00			
4	Maternal behavioral control	2.91	0.55	0.441 **	0.000	0.349 **	1.00		
5	Maternal psychological control	2.22	0.73	0.020	0.472 **	−0.113 **	0.052 *	1.00	
6	Mother-child relational quality	2.96	0.57	0.314 **	−0.089 **	0.424 **	0.594 **	−0.309 **	1.00
	**Wave 5**	**Mean**	**SD**	**1**	**2**	**3**	**4**	**5**	**6**
1	Paternal behavioral control	2.47	0.58	1.00					
2	Paternal psychological control	2.16	0.67	0.071 **	1.00				
3	Father-child relational quality	2.73	0.63	0.634 **	−0.237 **	1.00			
4	Maternal behavioral control	2.87	0.55	0.441 **	−0.020	0.321 **	1.00		
5	Maternal psychological control	2.20	0.70	0.010	0.450 **	−0.128 **	0.068 **	1.00	
6	Mother-child relational quality	2.94	0.57	0.316 **	−0.091 **	0.412 **	0.598 **	−0.272 **	1.00
	**Wave 6**	**Mean**	**SD**	**1**	**2**	**3**	**4**	**5**	**6**
1	Paternal behavioral control	2.45	0.59	1.00					
2	Paternal psychological control	2.17	0.71	0.057 *	1.00				
3	Father-child relational quality	2.73	0.62	0.659 **	−0.246 **	1.00			
4	Maternal behavioral control	2.85	0.53	0.463 **	0.010	0.308 **	1.00		
5	Maternal psychological control	2.20	0.72	−0.010	0.480 **	−0.124 **	0.010	1.00	
6	Mother-child relational quality	2.94	0.55	0.313 **	−0.099 **	0.383 **	0.614 **	−0.313 **	1.00

Note. * *p* < 0.05. ** *p* < 0.01.

## Appendix B

The 2-level model is presented as follows:

Level—1: Y*_ij_* = β_0*j*_ + β_1*j*_(Time) + β_2*j*_(Time^2^) + r*_ij_*

Level—2:

    β_0*j*_ = γ_00_ + γ_01_(parent gender) + γ_02_(student age) + γ_03_(student gender) + u_0*j*_

    β_1*j*_ = γ_10_ + γ_11_(parent gender) + γ_12_(student age) + γ_13_(student gender) + u_1*j*_

    β_2*j*_ = γ_20_ + γ_21_(parent gender) + γ_22_(student age) + γ_23_(student gender) + u_2*j*_
